# Bacteriophages to Control Multi-Drug Resistant *Enterococcus faecalis* Infection of Dental Root Canals

**DOI:** 10.3390/microorganisms9030517

**Published:** 2021-03-03

**Authors:** Mohamed El-Telbany, Gamal El-Didamony, Ahmed Askora, Eman Ariny, Dalia Abdallah, Ian F. Connerton, Ayman El-Shibiny

**Affiliations:** 1 Department of Microbiology and Botany, Faculty of Science, Zagazig University, Zagazig 44519, Egypt; Mohamedsamir9349@yahoo.com (M.E.-T.); gamse5@yahoo.com (G.E.-D.); ahmedaskora@yahoo.com (A.A.); emanariny@yahoo.com (E.A.); 2 Department of Endodontics, Faculty of Dentistry, Suez Canal University, Ismaïlia 41522, Egypt; daliadental82@gmail.com; 3 School of Biosciences, University of Nottingham, Loughborough LE12 5RD, UK; 4 Center for Microbiology and Phage Therapy, Zewail City of Science and Technology, 6th of October City 12578, Egypt

**Keywords:** *Enterococcus faecalis*, bacteriophage, phage therapy, root canal infection, multi-drug resistant

## Abstract

Phage therapy is an alternative treatment to antibiotics that can overcome multi-drug resistant bacteria. In this study, we aimed to isolate and characterize lytic bacteriophages targeted against *Enterococcus faecalis* isolated from root canal infections obtained from clinics at the Faculty of Dentistry, Ismalia, Egypt. Bacteriophage, vB_ZEFP, was isolated from concentrated wastewater collected from hospital sewage. Morphological and genomic analysis revealed that the phage belongs to the *Podoviridae* family with a linear double-stranded DNA genome, consisting of 18,454, with a G + C content of 32.8%. Host range analysis revealed the phage could infect 10 of 13 *E. faecalis* isolates exhibiting a range of antibiotic resistances recovered from infected root canals with efficiency of plating values above 0.5. One-step growth curves of this phage showed that it has a burst size of 110 PFU per infected cell, with a latent period of 10 min. The lytic activity of this phage against *E. faecalis* biofilms showed that the phage was able to control the growth of *E. faecalis* in vitro. Phage vB_ZEFP could also prevent ex-vivo *E. faecalis* root canal infection. These results suggest that phage vB_ZEFP has potential for application in phage therapy and specifically in the prevention of infection after root canal treatment.

## 1. Introduction

The main goal of endodontic treatment is thorough disinfection of the root canal system, followed by the application of an adequate three-dimensional tight seal to prevent bacterial re-colonization and promote the healing of periradicular tissues [1]. Post-treatment apical periodontitis is an infectious disease in root canal-treated teeth caused mainly by persistent intra-radicular infection. *Enterococcus faecalis*, a commensal Gram-positive microorganism, is frequently recovered from secondary persistent infections associated with root canal treatment failures [2,3]. Persistent infection can result in invasion of periradicular tissues with the subsequent development of apical periodontitis and /or diffused infection (cellulitis) [4]. The reason for post-treatment disease is largely attributed to the limitations of the present intra-canal disinfection protocols to combat intracanal *E. faecalis* infection [2,3]. The instrumentation is incapable of touching all the infected canal walls. Anatomically challenging areas include lateral canals, isthmi, apical ramifications, and dentinal tubules [5,6]. Moreover, attacking mature microbial biofilms with conventional antibiotics has limited efficacy, requiring much higher drug doses than usual. Bacteria residing in biofilms are frequently inaccessible to antibacterial agents and are afforded protection from the immune system. Biofilms therefore constitute a reservoir of bacteria that can be disseminated to establish chronic infections throughout the body [7]. *E. faecalis* is hard to eradicate, and especially from root canals [8,9]. *E. faecalis* lodged in recalcitrant biofilms are involved in the failure of endodontic treatments and antibacterial treatments [10,11]. Conventional antibacterial rinsing with chlorhexidine or sodium hypochlorite can enhance *E. faecalis* adaptation and growth in the presence of calcium hydroxide in intracanal dressings [12,13,14].

*E. faecalis* have a variety of virulence factors reported to be responsible for pathogenicity, including biofilm formation and the expression of surface adhesion components such as enterococcal surface protein (ESP), endocarditis-associated antigen, and capsular polysaccharides [15,16,17,18]. The expression of metalloprotease gelatinase E gene has been associated to higher biofilm production in *E. faecalis* recovered from root canal infections [19]. Cytolysin expression is also related to observations of pathogenicity in animal models of infection and is associated with mortality in human bacteremia patients [20,21,22].

Enterococci have emerged as a major cause of hospital-acquired infections that are difficult to treat due to intrinsic and acquired resistance to multiple antimicrobial compounds [23]. An increasing number of clinical cases are complicated by multi-drug resistant (MDR) bacteria, which has prompted a re-examination of phage therapy to combat and destroy these bacteria. There are several reasons why phage therapy can be more efficient than antibiotics in the eradication of MDR bacteria [24,25,26,27]: 1, the high specificity of phage for their host without impacting the normal microbiota [28], 2, the self-limiting nature of phage as it is multiplication is dependent on the presence and growth of the target bacteria [29], 3, the ability to refresh therapeutic phage preparations to retarget any bacteria surviving therapy to achieve eradication [30], 4, the efficacy of phages to penetrate and disrupt biofilms [31], and 5, the ease of isolating new phages from different sources and the low cost of developing phage preparations compared to the production of new antibiotics [32,33]. The isolation of new phages is important to widen the spectrum of phage activities and enhance applications to treat MDR bacteria emerging in therapy or decontamination applications [34,35].

The aims of this study were to isolate and characterize anti *E. faecalis* phage to evaluate phage lytic activity against *E. faecalis* biofilms, and to assess its therapeutic potential to prevent *E. faecalis* infection in dental treatments of root canals by using an ex vivo human root canal model.

## 2. Materials and Methods

### 2.1. Isolation and Identification of E. faecalis from Root Canal

#### 2.1.1. Patient Selection

One hundred and fifty patients were selected to join this study from the endodontic clinic at the faculty of Dentistry, Suez Canal University, Egypt, after the approval of Ethics Committee of the University, and a written informed consent obtained from all patients after explanation of the study procedure. All selected patients did not suffer of any chronic systemic illness and had not received antibiotic therapy within the previous 3 months. The patients selected had post-treatment apical periodontitis in a single rooted tooth. Teeth with an un-restorable coronal tooth structure, vertical root fractures, and periodontal pockets more than 4-mm deep were excluded from the study.

#### 2.1.2. Sampling Procedures

Strict aseptic technique was applied during sampling. Scaling was undertaken to remove any plaque or calculus. The tooth was polished with pumice and 3% hydrogen peroxide. Rubber dam isolation was performed; caries and/or coronal restorations were removed with sterile high-speed burs. Sterile saline was used as coolant and high suction was applied. The operative field, including the tooth, clamp, and surroundings, were disinfected using 30% (*v*/*v*) hydrogen peroxide and 2.5% (*w*/*v*) NaOCl. Access opening was achieved with new sterile burs, after which the operative field including the pulp chamber was cleaned and disinfected once again. NaOCl was neutralized with 5% (*w*/*v*) sodium thiosulfate (Sigma-Aldrich, St Louis, MO, USA), and sterility control samples (SR1) were taken from the tooth surface using a sterile Omni Swab with an ejectable head (Whatman FTA, Sigma-Aldrich). The swab was transferred to a cryotube containing Tris-EDTA buffer (10 mM Tris-HCL, 1 mM EDTA, pH 7.6) (Sigma-Aldrich) and immediately placed in a Labtop cooler (−20 °C Nalgene Labtop cooler, Sigma-Aldrich) or directly to a freezer (−80 °C). Samples in the Labtop cooler were later transferred to a freezer. Sterility control samples were required to be uniformly negative after culturing on Bile Esculin Azide agar selective medium (Sigma-Aldrich) for the detection of *E. faecalis*. The coronal two thirds of the root fillings were mechanically removed with H-type hand files (Dentsply Maillefer, Ballaigues, Switzerland) and Fanta blue rotary orifice opener files (Fanta, Plano, TX, USA). The canal was filled with sterile saline solution with care to not overflow; a new sterile #15 K-file (Dentsply Maillefer) was inserted 1-mm short of the apical foramen with the help of the Root ZX electronic apex locator (J Morita Corp, Tokyo, Japan) with confirmation by radiograph; then a gentle filing motion was applied. Successive larger endodontic files were used to engage the root canal filling material. On withdrawal from the canal, the instrument was cut with a sterilized wire cutter, and the fragment with attached root filling material was put in a cryotube. In addition, the root canal walls were filed with sterile saline without suction, and the entire canal content was absorbed onto 3 sterile paper points that were inserted to the full working length that left for about 1 min. The paper points were then transferred to Tris-EDTA buffer. Apical preparation was completed to the working length with Fanta blue rotary files (Fanta, USA) and hand nickel-titanium files (NitiFlex, Dentsply Maillefer) with sterile saline irrigation. Master apical files ranged from #40 to #60 depending on both the root anatomy and the initial diameter of the root canal was reached. Other samples were taken from the canal lumen using sterile paper points as described earlier. Samples (paperpoints) were collected in sterile universal tubes containing 5 mL saline and 1% (*w*/*v*) yeast extract and transported to laboratory and immediately diluted before culturing on Bile Esculin Azide agar medium (Sigma-Aldrich, St Louis, MO). The root canal was then irrigated with 2.5% (*w*/*v*) NaOCl using ultrasonic activation and 2% cycloheximide (*w*/*v*) as final rinse [36,37]. The canal was dried, and obturated with gutta-percha and AH Plus sealer (Dentsply) using the cold lateral compaction technique [38]. The tooth access was sealed with composite restoration, and a final radiograph was taken. Black colonies were collected for further biochemical and molecular identification at Animal Health Research Institute, Dokki, and Giza, Egypt. Stock cultures were stored in LB broth containing 20% (*v*/*v*) glycerol at −20 °C.

### 2.2. Biochemical Identification of E. faecalis

Presumptive colonies of *E. faecalis* were Gram stained and tested for catalase. Identification was confirmed using API 20 STREP strip systems (Biomerieux, Cairo, Egypt), according to manufacturer’s recommendations, and the VITEK system, according to manufacturer´s recommendations at the Biotechnology unit, Animal Health Research Institute, Dokki, Giza, Egypt.

### 2.3. Antimicrobial Susceptibility Test

Antimicrobial susceptibility testing was performed using the minimal inhibitory concentration (MIC) method by using the VITEK system, located at the Biotechnology unit, Animal Health Research Institute, Dokki, Giza, Egypt. MICs of ampicillin, penicillin G, ciprofloxacin, levofloxacin, erythromycin, quinupristin⁄dalfopristin, linezolid, vancomycin, tetracyclin, tigecycline, nitrofurantoin, gentamycin and streptomycin were interpreted according to Clinical Laboratory Standard Institute (CLSI) guidelines, 2015 [39].

### 2.4. PCR Analysis of E. faecalis Genomic DNAs

DNA extraction from samples was performed using the QIAamp DNA Mini kit (Qiagen, Düsseldorf, Germany, GmbH) with modifications from the manufacturer’s recommendations. Briefly, 200 µL of the sample suspension was incubated with 20 µL of proteinase K and 200 µL of lysis buffer at 56 °C for 10 min. After incubation, 200 µL of 100% ethanol was added to the lysate. The sample was then centrifuged and washed following the manufacturer’s recommendations. Nucleic acid was eluted with 100 µL of elution buffer provided in the kit. PCR amplification of 16S rRNA gene was performed as described previously [37] using the forward primer 5′-GTTTATGCCGCATGGCATAAGAG-3′ and reverse primer 5′-CCGTCAGGGGACGTTCAG-3′ (Midland Certified Reagent Company, Midland, TX, USA). Six oligonucleotide primer pairs (Midland Certified Reagent Company) were used to amplify the genes encoding the aggregation substances protein (*asa1*), gelatinase (*gelE*), cytolysin (*cylA*), surface protein (*esp*), collagen binding protein (*ace*) and surface antigen (EF3314) for *E. faecalis*, as described previously [40,41]. The primer sequences and expected amplicon sizes are listed in Supplementary Table S1. PCR was carried out in a 25 µL reaction containing 12.5 µL of EmeraldAmp Max PCR Master Mix (Takara, Japan), 1 µL of each primer (20 pmol), 4.5 µL of water, and 6 µL of DNA template (30 ng). The reactions were performed in an Applied Biosystems 2720 thermal cycler (Biometra, Göttingen, Germany). The products of PCR were separated by electrophoresis on 1.5% agarose gel (Applichem GmbH, Germany) in 1x TBE buffer (10.78 g/L Tris buffer, 5.5 g/L Boric acid, 0.82 g/L EDTA, pH 8.3) at room temperature using gradients of 5 V/cm. For gel analysis, 20 µL of the products was loaded in each gel slot. A Gelpilot 100 bp Ladder (Qiagen GmbH, Germany) was used to determine the fragment sizes. The gel was photographed by a gel documentation system (Alpha Innotech, Biometra) and the data was analyzed using associated software [37].

### 2.5. Isolation of Bacteriophages Infecting E. faecalis

Bacteriophages were isolated from different sewage water samples obtained from Zagazig City, Egypt by the enrichment technique [42]. The sewage samples were clarified by centrifugation at 6000× *g*. for 20 min, after particle removal by paper filters, the water sample was filtrated through 0.45-μm pore membranes. Then, a 100 mL of the filtrate was added to an equal volume of 2× TSB (Tryptic Soy Broth; Oxoid, UK) medium and 1 mL of log phase culture of different isolated *E. faecalis* (3 × 10^8^ CFU/mL) and incubated in a shaking incubator 120 rpm at 37 °C for 24 h. The culture was then centrifuged at 6000× *g*. for 10 min, treated with chloroform and the supernatant filtered through 0.45 μm pore membranes to remove the bacterial culture, and the supernatant was used as a source of possible phages to be detected on the propagative strain. Bacterial lawns of *E. faecalis* were poured over the surface of TSA (Tryptic Soy Agar; Oxoid, UK) plates using the double agar overlay technique, as described by Adams [42]. Briefly, 100 μL of a mid-exponential phase of bacterial culture was added to 3 mL semi-solid TSA and poured over solid TSA agar plates. After drying, 15 μL droplets of the possible phage source previously prepared were spotted onto the lawns and left to dry. The plates were incubated overnight at 37 °C and checked for the presence of lysis zones.

### 2.6. Bacteriophages Purification and Propagation

Bacteriophages were propagated and purified from single plaque isolates according to previous studies [42,43]. Phage isolates were purified by picking three successive single plaques using a sterile Pasteur pipette until homogenous plaques were obtained. Briefly, a single plaque was picked and put into 5.0 mL nutrient broth containing 100 μL of bacterial host and then incubated at 37 °C under shaking condition with 1200 rpm. After incubation, chloroform was added and the phage-host mixture centrifuged at 6000× *g*. for 10 min and the supernatant was filtered through a sterilized 0.45-μm Millipore filter (Steradisc, Kurabo Co., Ltd. Osaka, Japan) before filtration with 0.22-μm Millipore filter. The purified phage was stored at 4 °C.

### 2.7. Determination of Host Range and Cross Infectivity of the Isolated Bacteriophages

The isolated phage was investigated for host range specificity and lysis efficiency. Bacterial lawns off different bacterial species were propagated on TSA plates as described before, and 10-μL droplets of phage were spotted on the lawns. The plates were incubated at 37 °C for 24 h and checked for the presence of plaques. To assess the host-range of the isolated phage, *Streptococcus mutans, Enterococcus gallinarum, Enterococcus faecium, Escherichia coli, Pseudomonas aeruginosa*, and *Staphylococcus aureus* were each inoculated on double-layered agar plates containing the appropriate medium, and streak tests were performed.

### 2.8. Efficiency of Plating (EOP)

Bacteriophage vB_ZEFP was tested in triplicate over eight decimal dilutions against all the susceptible bacterial strains lysed in the spot assays, as previously described [44]. Conditions of these experiments were similar to the conditions of the spot tests. Thus, 200 μL of all bacterial isolates in Log-phase bacteria were added to top agar, and different dilutions of phages were spotted on the surface of petri dishes containing the bacterial host. The plates were incubated overnight at 37 °C and the EOP was estimated as the average PFU on target bacteria/average PFU on host bacteria.

### 2.9. Determination of the Frequency of Bacteriophage Insensitive Mutants

The frequency of the emergence of bacteriophage insensitive mutants (BIMs) was estimated. Phage vB-ZEFP was mixed with bacterial host strains that were confirmed to be susceptible to the bacteriophage at an MOI of 10. After 10 min of incubation at 37 °C, the suspension was serially diluted and spotted using double agar overlay plaque assays. Plates were incubated overnight. The survivors of phage infection were calculated correspondingly by dividing bacterial viable counts remained after phage infection by initial viable counts. Experiments were conducted in triplicate.

### 2.10. Examination of Phage Morphology by Electron Microscopy

Bacteriophage particles were stained with Na-phosphotungstate or uranyl acetate before observation in a Hitachi H600A electron microscope, as described previously [43]. A drop of phage suspension at high titer (10^11^ PFU/mL) was placed on 200 mesh copper grids with carbon-coat formvar films, and any excess drawn off by absorption to filter paper. A saturated solution of Na-phosphotungstate or uranyl acetate was then placed on the grids and the excess drawn off as before. Specimen was examined with an electron microscope in a Hitachi H600A electron microscope, Faculty of Agriculture, Mansoura University.

### 2.11. Assessment of Phage Lytic Activity in Biofilms

The activity of isolated phage against established biofilms of *E. faecalis* 4 was determined using the microtitre plate assay as previously described [45]. Briefly, the bacterial culture broth was diluted 1:100 in LB. (Oxoid, UK) and loaded into the wells of untreated 96-well polystyrene flat-bottom microtitre plate (Costar, Corning Inc., Corning, NY, USA). The plates were then incubated at 37 °C for 24 h under gentle shaking to allow biofilm formation. Unattached planktonic cells were carefully removed by washing twice in 0.9% (*w*/*v*) NaCl. Using MOIs 0.1, 1, and 10, phage was diluted in LB and added to each well 1 day after the biofilm established, whilst saline solution was added to other wells as a control. The plates were incubated for 24 h at 37 °C. The biofilms were washed with 0.9% NaCl, and the biofilm biomass quantified by staining with crystal violet (1% *w*/*v*) for 20 min. Following washing with 0.9 NaCl solutions, the crystal violet was solubilized in ethanol (95%) and then, the absorbance was measured using an ELISA plate reader at OD_600._

### 2.12. One-Step Growth Curve

One step growth experiments were performed as previously described [46]. Briefly, E. faecalis strains were grown at OD = 0.2 (~10^8^ CFU/mL) and infected with bacteriophage at MOI of 0.1 and then, allowed to adsorb for 10 min at room temperature. The mixture was centrifuged, and the pellets re-suspended in 10 mL of LB medium and incubated at 37 °C. Samples (200 μL each) were obtained at 5-min intervals and divided into two volumes of 100 μL. The first sample (without the addition of chloroform) was immediately diluted and plated for phage titration to enumerate the phage particles released from the infected bacterial cells, while chloroform was added to the other sample at a concentration of 1% (*v*/*v*) to release the intracellular phages in order to determine the eclipse period.

### 2.13. Bacteriophage Temperature and pH Stability

The temperature stability of phage was estimated at different temperatures (40, 50, 60, 70, 80, 90, and 100 °C) over 1 h. Samples were obtained at 10 min intervals, and immediately diluted and plated for phage titration [47]. The stability of phage at different pH values (2, 4, 6, 8, 10, 12, and 13) was determined using SM phage buffer (5.8 g of NaCl, 1.2 g of MgSO_4_, 50 mL of 1 M Tris-HCl pH 7.5 with 0.1 g of gelatin in 1000 mL of deionized water) and the pH adjusted by the addition of 1 M HCl or 1 M NaOH solutions. After incubation of the mixtures at 4 °C overnight, the residual phage activity was determined by plaque assay technique, as previously reported [46].

### 2.14. Isolation and Characterization of Nucleic Acids from Phage Particles

Phage DNA was extracted using a phenol/chloroform method [48]. Briefly, 200 μL of purified suspension was treated with lysis buffer (0.5% (*w*/*v*) SDS, 100 μg/mL proteinase K, 20 mM EDTA) for 1 h at 56 °C. An equal volume of phenol: chloroform: isoamyl alcohol (25:24:1) was added to remove proteinaceous material. Extraction was repeated twice, and between each step the upper aqueous phase layer was transferred into a new phage-lock gel tube and gently vortexed. The mixture was centrifuged at 10,000× *g* at 4 °C for 5 min. The supernatant was transferred to a new sterile 1.5 mL Eppendorf tube and DNA was precipitated by adding an equal volume of isopropanol and the mixture allowed to stand at −20 °C overnight. The DNA pellet was centrifuged at 10,000× *g* at 4 °C for 20 min and washed twice with 75% (*v*/*v*) ethanol and suspended in deionized water before storage at −20 °C. The DNA was digested with restriction enzymes BamHI, EcoRI and HindIII according to the supplier’s instructions (Takara Bio Inc., Kusatsu, Shiga, Japan). Conditions and buffers were chosen according to the manufacturer’s instructions. The incubation times for the restriction enzymes were 2 to 4 h. The digested DNA sample was analyzed by electrophoresis at 100 V in a 1.0% (*w*/*v*) agarose gel stained with ethidium bromide using a DNA ladder as marker.

### 2.15. Phages Genome Sequencing

DNA sequencing was performed using the Illumina MiSeq platform. Library preparation of vB_ZEFP genomic DNA followed the Illumina Nextera tagmentation protocol (Illumina, Cambridge, United Kingdom) and the library sequenced using the Illumina v3 sequence cassette for 600 cycles on the MiSeq. The data consisted of 1.1 and 3.4 million paired-end sequence reads of 60 to 250 bp in length for vB_ZEFP. Initial processing of the raw data and de novo assembly was performed using CLC Genomics Workbench version 11.0.1 (Qiagen, Aarhus, Denmark). The assembled reads yielded double-stranded linear DNA genome of 18,454 bp for vB_ZEFP. Gene predictions were made using PHASTER [49], HHpred [50] and BLAST (Non-redundant NCBI databases; [51]) to identify putative open reading frames (ORFs), followed by manual curation and polishing with Artemis [52]. The sequence is available under the GenBank accession number MT747434. Phylogenetic analysis was performed from pairwise alignments generated by BLASTN [51] using the fast minimum evolution method of Despr and Gascuel [53].

### 2.16. Ex Vivo Human Root Canal Model

#### 2.16.1. Tooth Selection and Preparation

Eighty extracted single-rooted teeth were collected from an outpatient clinic, Faculty of Dentistry, Suez Canal University, after the approval of the faculty ethics committee. Only teeth with a type I root canal were included in the study and teeth with open apexes, cracks or apical ramifications were excluded. Teeth were cleaned from any debris by scaler and disinfected with 2.5% (*w*/*v*) NaOCl. The crown was removed using sterile rotary disk associated with coolant at the cemento-enamel junction level. The roots were painted with two layers of nail varnish to seal the root periphery except the apical foramen.

Standard endodontic access cavity was undertaken using a sterile bur with coolant. Patency was checked with #15 k-file (Micro Mega, Besancon, France) to the working length (1 mm from the apex). Canal orifice was opened and flared using Fanta rotary orifice opener files (Fanta, USA). All teeth were autoclaved for sterilization and kept in sterile laminar flow.

#### 2.16.2. Tooth Contamination and Obturation

Canals were treated by injection of *E. faecalis* suspensions (200 μL from a culture with an OD_600_ of 0.1) in the coronal orifice of each tooth. Teeth were randomly divided into four groups (*n* = 20). Shaping of the contaminated canals was achieved using K-files (Micro Mega, Besancon, France) and Fanta rotary files (Fanta, USA), whilst cleaning was performed using the following irrigation protocols for each group:

Group A: saline irrigation as a negative control
Group B: 200 µL of phage (10^8^ PFU/mL) irrigation
Group C: 2.5% NaOCl and 200 µL of phage (10^8^ PFU/mL) irrigation
Group D: 2.5% NaOCl and 17% EDTA irrigation

After the third rotary file (AF3 25/06) shaping, the canals were re-contaminated with an *E. faecalis* suspension. Final cleaning and shaping were performed up to AF4 35/04 followed by K-files (#40) as a master apical file coupled with the corresponding irrigation protocol, EDTA cream (Micro Mega) was used as a final rinse. The canals were obturated using lateral condensation technique with gutta-percha and an endodontic sealer (AH plus; Dentsply, Germany). Bacterial leakage was assessed using a two-chamber bacterial leakage model using LB. as previously reported [54,55]. The culture turbidity was estimated every 24 h, optical density was measured, and samples were plated to determine the viable count (CFU/mL).

### 2.17. Statistical Analysis

Analytical statistics were calculated using ANOVA with GraphPad PRISM software. A *p*-value of 0.05 or less was considered statistically significant in all cases.

## 3. Results

### 3.1. Prevalence of E. faecalis in Root Canals

**Figure 1:**
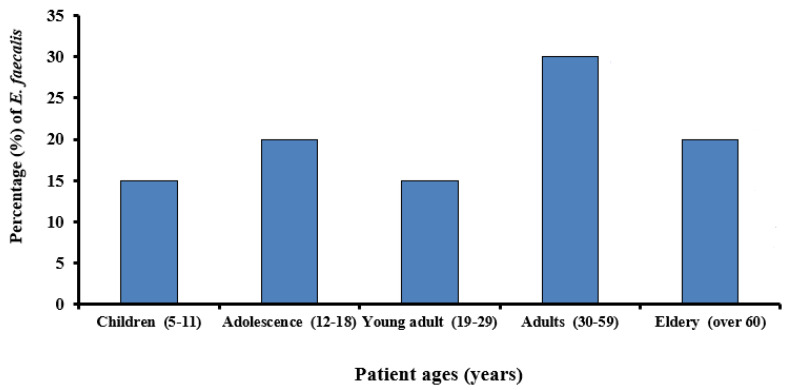
Age distribution of oral carriers of *E. faecalis*.

A total of twenty (13.3%) *E. faecalis* confirmed isolates were recovered from one hundred and fifty samples collected from oral cavity at the clinics at the faculty of Dentistry, Ismailia, Egypt. Figure 1 shows the age distribution of individuals positive for oral *E. faecalis.*

### 3.2. Sensitivity of E. faecalis to Antibiotics

Bacterial isolates were tested for their susceptibilities to 13 antibiotics prescribed in human medicine. The susceptibility or resistance of isolates to antibiotics was determined by determining the minimal inhibitory concentration (MIC) using the VITEK system. The antimicrobial susceptibility profiles of *E. faecalis* (Table 1) demonstrated marked differences in the frequencies of antibiotic resistance. *E. faecalis* was sensitive to linezolid, vancomycin and tigecyclin, but resistant to benzylpencillin, ampicillin, gentamycin, streptomycin, ciprofloxacin, levofloxacin, erythromycin, tetracycline, nitrofurantoin, and streptgramins. This study indicated that the majority (16/20) of *E. faecalis* isolated from root canal infections were resistant to multiple antibiotics. For example, EF 2 was resistant to 7 out of 13 antibiotics (54%), while EF 4 is resistant to 10 out of 13 antibiotics (77%).

### 3.3. Survey of Virulence Determinants in E. faecalis Isolates

A survey of six virulence genes of *E. faecalis* isolate No. 4 by PCR amplification (Supplementary Figure S1) revealed the presence of the genes encoding the collagen binding protein (*ace*), aggregation substances protein (*asa1*), and gelatinase (*gelE*). However, no amplification products were detected for the genes encoding cytolysin (*cylA*), surface protein (*esp*) and surface antigen (EF3314).

### 3.4. Isolation of Bacteriophages

Bacteriophage specific to MDR *E. faecalis* was isolated from four sewage samples collected from Zagazig University hospital, Sharkia Province, Egypt using the spot test technique on double layer agar. Of the 4 samples collected only one showed phage activity against *E. faecalis* (isolates no. 1, 2, 4, 5, 7, 8, 9, 10, 12 and 13) as the host. Selection of the vB_ZEFP bacteriophage was undertaken upon serial passage according to their ability to lyse a broad range of *E. faecalis* isolates, generate reproducible clear zones of lysis, and their capability to replicate to produce high titers on the selected host over time. The phage was plaque purified and amplified by plate lysis in preparation for characterization.

### 3.5. Morphological Characterization of Phages by Electron Microscopy

**Figure 2:**
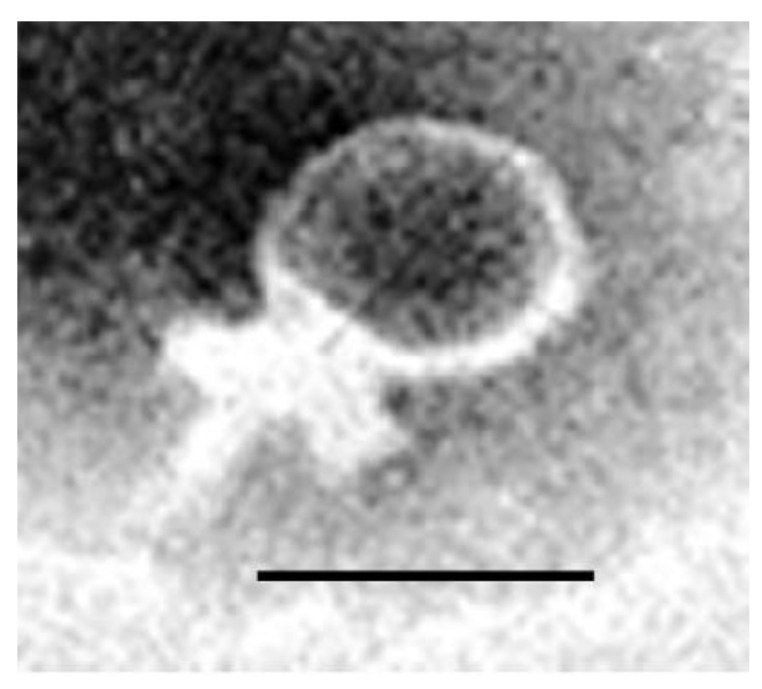
Transmission electron micrograph of phage vB_ZEFP: The phage exhibits a short non-contractile tail indicative of the *Podoviridae* family. The scale bar represents 50 nm.

The morphology of vB_ZEFP was characterized using TEM after staining with uranyl acetate. Based on electron micrographs (Figure 2), the vB_ZEFP phage has icosahedral heads with short non-contractile tail, morphology typical of phage belonging to the *Podoviridae* family. Phage vB_ZEFP had mean head dimensions of 43.4 ± 2.1 × 41.1 ± 1.8 nm, while tail length was calculated to be 20 ± 0.5 nm.

### 3.6. Host Range and EOP of vB_ZEFP Phage

The host range of the isolated phage was determined using thirteen *E. faecalis* isolates, and with laboratory strains of *Enterococcus faecium*, *Enterococcus gallinarium*, *Streptococcus mutans, E. coli*, and *Staphylococcus aureus* (Supplementary Table S2). The results showed that the vB_ZEFP phage could infect 10 of 13 *E. faecalis* isolates used as hosts in this study, indicating broad host coverage of the oral *E. faecalis* isolates. The results showed no lytic effects on any of the alternative host species tested. The ability of vB_ZEFP phage to lyse multiple *E. faecalis* was further assessed by examining the efficiency of plating (EOP). The selected phage exhibited EOPs ≥ 0.5 for all *E. faecalis* examined isolates.

### 3.7. Frequency of Bacteriophage Insensitive Mutants

The development of BIMs could compromise the long-term effectiveness of using phage as therapeutic agents. Assays were performed in vitro to determine the frequency at which BIMs emerged at 37 °C. BIMs were recovered following high multiplicity infections (10) of host bacteria with bacteriophage vB-ZEFP at 37 °C. The host survival frequency was determined to be low at 4 × 10^−7^ ± 3 × 10^−7.^

### 3.8. Bacteriophage Activity Against E. faecalis Established in Biofilms

**Figure 3:**
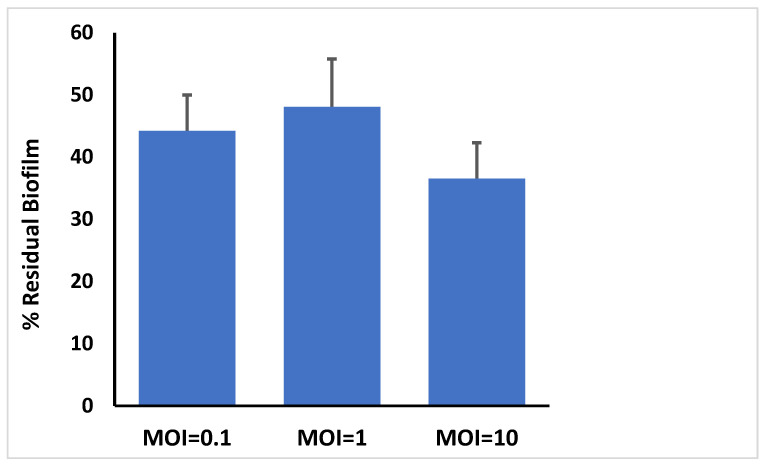
vB_ZEFP phage mediated reduction of *E. faecalis* biofilms. Microtitre plates containing preformed biofilms were treated either with phage for 24 h. Biofilm contents were estimated by OD measurement of ethanol solubilized crystal violet-stained material and reported as residual biofilm. MOI indicates the multiplicity of infection of the phage with respect to the initial *E. faecalis* viable count. All phage treatments reduced biofilm contents compared to the carrier treated controls (*p* < 0.003).

*E. faecalis* isolate 4 was allowed to form biofilms in microtitre plates and challenged with phage at different MOIs. The wells were evacuated after 24 h and stained with crystal violet before OD measurement of ethanol solubilized crystal violet using an ELISA plate reader (Figure 3). The results showed that the phage preparation significantly reduced the crystal violet stainable biofilm content compared to control (*p* < 0.003). The highest MOI of 10 produced the greatest reductions, exhibiting a significantly greater reduction compared to MOIs 0.1 and 1 (*p* < 0.004).

### 3.9. Phage Growth Characteristics

**Figure 4:**
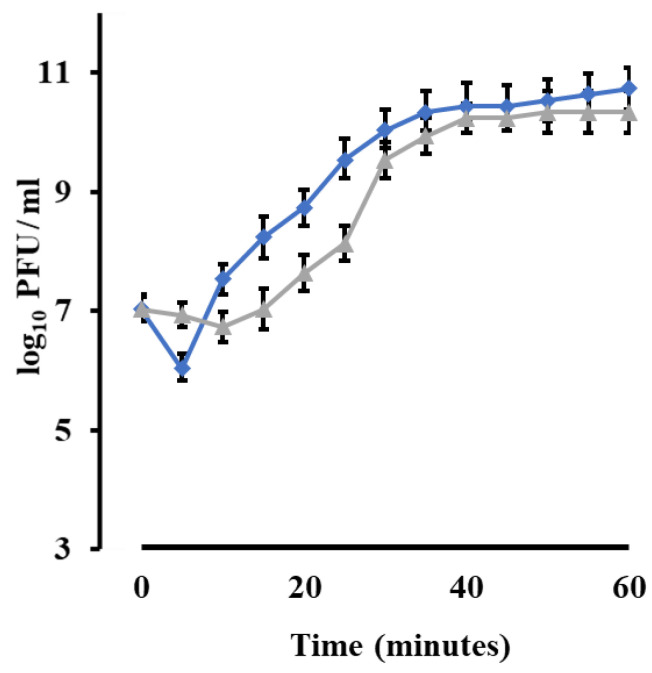
Single step growth curve. The grey line represents nascent phage without chloroform addition (PFU/mL), while the blue line represents phages released after chloroform addition (PFU/mL). The phage titers are recorded as means and the error bars are ± standard deviation of three replicates.

Burst size and latent period were determined for vB_ZEFP phage by performing one-step growth curves (Figure 4). Chloroform aids new phage particles to free from bacterial cell wall. The phage produced estimated burst size of 110 PFU per infected cell with a latent period of 10 min.

### 3.10. Bacteriophage Temperature and pH Stability

**Figure 5:**
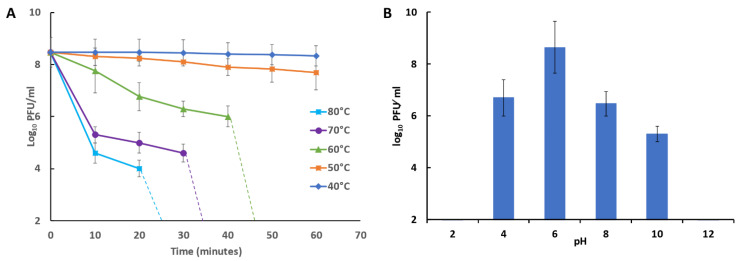
(**A**) Temperature and (**B**) pH stability of *E. faecalis* phage vB_ZEFP.

The stability of isolated phage at different temperature and pH values was investigated. The results indicated that the phage titers were stable at approximately 10^8^ PFU⁄ mL for 50 min at temperatures of 40 °C and 50 °C. At 60 °C, the phage titer decreased after 40 min to 10^6^ PFU/mL. The phage titer fell to 10^5^ PFU/mL after 30 min at 70 °C. (Figure 5A) and fell to 10^4^ PFU/mL after 20 min at 80 °C. The results revealed activity and stability of the phages over a broad pH range of 4 to 10 with the optimum titer at pH 6. However, post exposure to pH 2 and pH 12 the phage titer was undetectable. (Figure 5B).

Panel A shows the thermal stability of phage vB_ZEFP exposed to temperatures 40 to 80 °C and the phage titers measured at 10-min intervals. The dashed lines indicate where the phage titers fell below the level of detection (2 log_10_ PFU/mL). Panel B shows pH on stability of the vB_ZEFP phage. Phage suspensions were incubated at pH indicated for 16 h at 30 °C. Phage titers are recorded as means ± standard deviation.

### 3.11. Phage Genome

**Figure 6:**
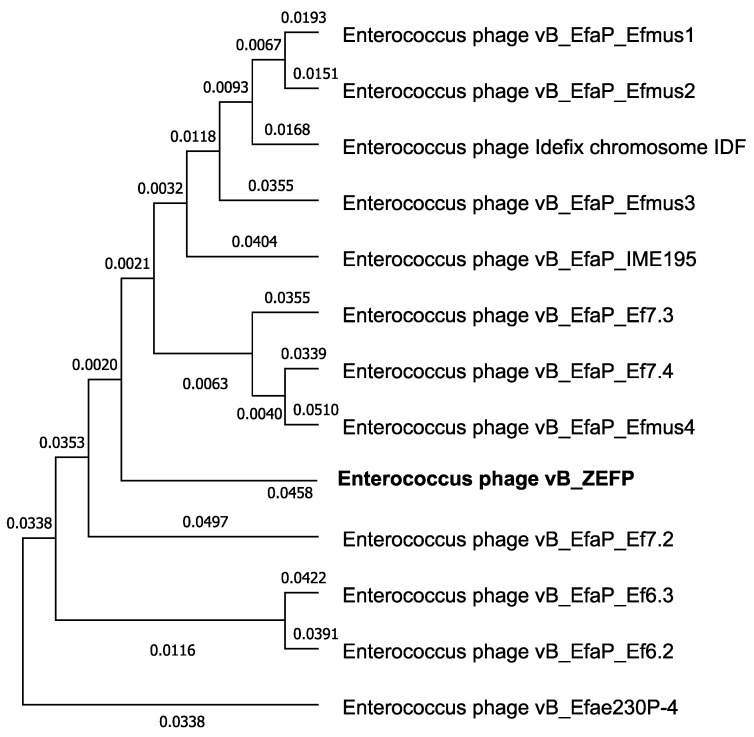
Phylogenetic relationships between *Picovirinae* phages that infect *Enterococcus.*

Gel electrophoresis of the genomic DNA of vB_ZEFP phage indicated that the phage genome to be approximately 18 kb with similar size products generated upon HindIII restriction endonuclease digestion. DNA sequencing of the vB_ZEFP phage DNAs enabled de novo assembly and accurate size determination of linear genomes of 18,454 bp (Genbank accession MT747434) with G + C contents of 32.8%. BlastN searches of the non-redundant database at NCBI revealed the phage genome to exhibit 86–92% nucleotide identity with a group of enterococcal phages classified in the family *Podoviridae*, subfamily *Picovirinae*. Figure 6 presents the phylogenetic relationships between the enterococcal *Podoviridae*. The genome sequence contained 28 open reading frames, of which 15 could be ascribed putative functions on the basis of BLASTP, Phaster, and HHPred database searches, whilst 13 remained as hypothetical proteins. Reading frames for which putative functional information could be ascribed to the products appear in Table 2.

Un-rooted phylogenetic tree of Enteroccocal phage genome sequences that can be ascribed to the *Picovirinae* subfamily of the *Podoviridae*. Phage vB_ZEFP is marked in bold and positioned central to the clade. Genome sequences were pairwise aligned with BLASTN and the tree constructed using the fast minimum evolution method of Despr and Gascuel [53] with a maximum sequence difference of 0.75 to maximize the accuracy of the groupings.

### 3.12. Ex Vivo Human Root Canal Model

**Figure 7:**
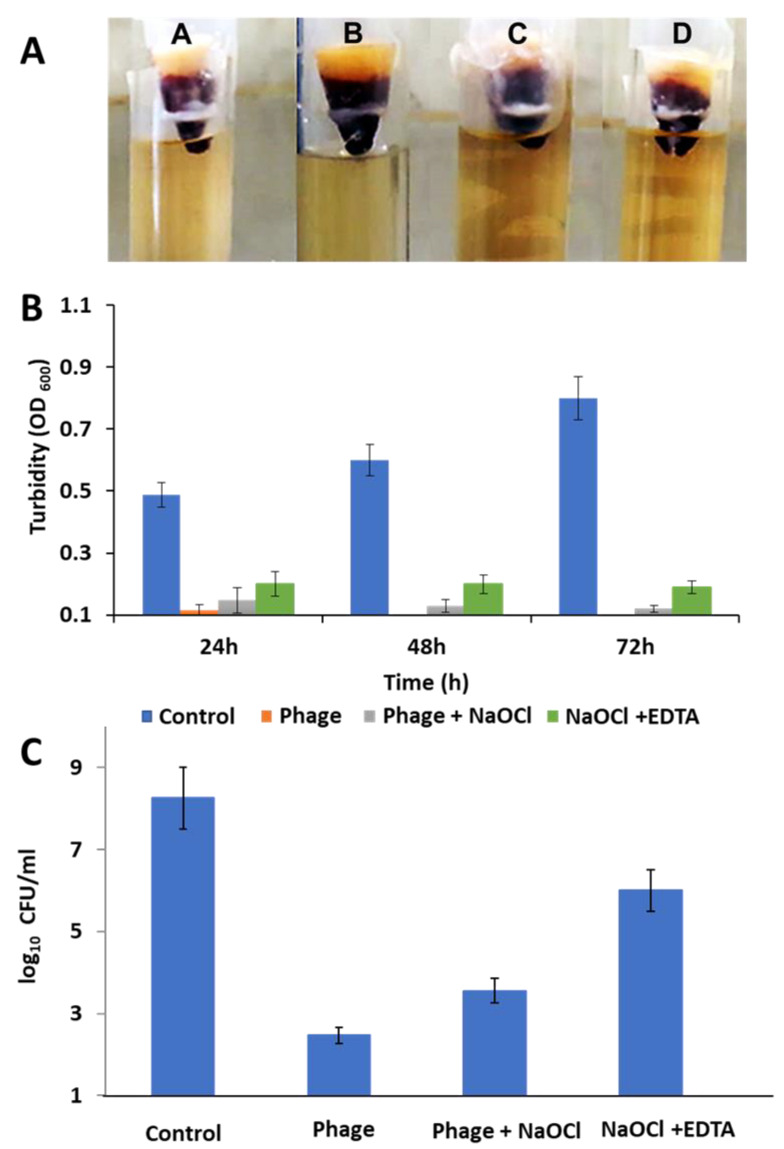
Irrigation efficiency of *E. faecalis* infected root canals. Panel (**A**) shows the two-chamber leakage model from root canals infected with *E. faecalis* isolate 4 and irrigated with either A. saline solution (control); B. phage treatment (vB_ZEFP 10^8^ PFU/mL); C, phage plus 2.5% NaOCl; D. 2.5% NaOCl and 17% EDTA. Panel (**B**) shows culture turbidity measured every 24 h at OD600. Panel (**C**) shows viable counts after 72 h. Error bars indicate ± standard deviation of the mean.

Eighty *E. faecalis* (isolate 4) infected root canals were irrigated with four irrigants (*n* = 20) in order to determine the most effective procedure against *E. faecalis*. The irrigants were phage (vB_ZEFP), phage and NaOCl, NaOCl and EDTA, and a saline solution as a control (Figure 7A). The efficacy of the irrigant was evaluated by measuring the turbidity in LB. (OD) arising due to residual bacterial leakage. All treatments showed a reduction in the OD compared to the control (*p* < 0.02; Figure 7B), which was confirmed by viable count at the experimental end point of 72 h (*p* < 0.03; Figure 7C). Based on the irrigants ability to suppress *E. faecalis* growth, we concluded that irrigation with phage was more efficient in preventing leakage from the root apex compared to the other treatments. Irrigation with phage and NaOCl was also better than NaOCl alone with the lowest viable counts recorded for phage treated groups: phage only (2 × 10^2^ CFU/mL), phage with NaOCl (3 × 10^4^ CFU/mL), and NaOCl and EDTA (3 × 10^6^ CFU/mL). In the future, phage may be used with NaOCl to prevent root canal treatment failure instead of using high concentrations of NaOCl, which can affect the oral microbiota and the human immune system.

## 4. Discussion

There is an increasing number of antibiotic-resistant *E. faecalis* strains encountered in clinical settings, and the protection afforded by biofilm formation make treatments such as antiseptic rinses or antibacterial dressings increasingly ineffective. To control *E. faecalis* necessitates alternative methods to be developed and executed [56]. The use of lytic bacteriophages is a promising application under consideration to fight multidrug resistant *E. faecalis* strains and their biofilms [55]. In this study, we have isolated *E. faecalis* from root canal infections and examined their antibiotic susceptibility patterns. Only 3 of 20 confirmed isolates were sensitive to all the antibiotics tested. Multi-drug resistance was encountered in 15 of the 20 isolates with one strain resistant to 10 antibiotics out of 13 tested. The incidence of multi-drug resistance may be due to the general misuse of antibiotics. We have investigated the presence of genes involved in *E. faecalis* virulence and the biofilm formation in the isolates from dental root canal samples of patients with chronic periodontitis. These revealed the presence of genes encoding the collagen binding protein (*ace*), aggregation substances protein (*asa1*), and gelatinase (*gelE*), which several studies have ascribed roles in adhesion and biofilm formation for *E. faecalis* [57,58].

In this study, a phage specific to *E. faecalis* was isolated from four sewage samples and designated as vB_ZEFP. Transmission electron microscopy showed that the phage belonged to the *Podoviridae* family with an icosahedral head and short tail. The genome sequence was consistent with this classification and exhibited similarity to other phages recently reported for *E. faecalis* [59,60] and falls within orthocluster VI in the comprehensive evaluation of the available *E. faecalis* phage genome sequences by Bolocan et al., which they concluded are suitable for phage therapy applications [61]. Bacteriophage host ranges impact on their utility for phage therapy. A host range restricted to one species is desirable, because it prevents collateral damage leaving the remainder of the host microbiome intact [62]. Phage vB_ZEFP was specific for *E. faecalis*, but could infect 10 out of 13 different strains isolated from root canals. Phage vB_ZEFP is highly productive with a burst size of 110 per infected cell and a latent period of 10 min, which makes it suitable for phage therapy applications. The efficiency of plating was calculated as the ratio between bacteriophage titer on clinical isolate to bacteriophage titer on the strain used to isolate and replicate the bacteriophage. The selected phage exhibited EOPs ≥ 0.5 for all *E. faecalis* isolates examined. This is indicative that vB_ZEFP phage is able to efficiently infect multiple target strains to reduce the numbers of viable bacteria over a wide host range. The phage was stable over a broad pH range (4 to 10) and at temperatures up to 50 °C with residual activity detectable (4 log_10_ PFU/mL reduction) after 20 min exposure at 80 °C. *E. faecalis* strains form biofilms, which increases their resistance to antibacterial treatments that include traditional antibacterial rinsing solutions such as sodium hypochlorite and chlorhexidine and enable the bacterium to grow in the presence of calcium hydroxide [63,64,65]. We tested the ability of the vB_ZEFP phage to reduce pre-formed bacterial biofilms. The results showed that the ability of phage to reduce the crystal violet stainable biofilm content. The greatest reduction of *E. faecalis* biofilm was observed when the bacterial host was treated with a phage for the MOI of 10 in comparison with the carrier-treated control.

Genomic analysis confirmed that vB_ZEFP phage belongs to the *Podoviridae*. The phage contains a linear, double-stranded DNA genome of 18,454 bp with 28 open reading frames. BLASTn analysis revealed that vB_ZEFP phage shares 92.51% identity with vB_EfaP_IME195 *E. faecalis* phage, which has a linear double-stranded DNA genome of 18,607 bp annotated with 27 coding sequences [59]. Our phage differs from IME-EF1, HEf13 and EFDG1 reported to infect oral *E. faecalis*. IME-EF1 and HEf13 belong to the *Siphoviridae* family with linear double-stranded DNA genomes of 57,081 and 57,811 nucleotides respectively [66,67]. EFDG1 has a DNA genome of 149,589 bp and is classified in the *Spounavirinae* subfamily of the *Myoviridae* [54]. EFDG1 was also reported to reduce the content of 2-week pre-formed biofilms, which is indicative of a general advantage of phage therapy over conventional antibiotic treatments against bacteria embedded in biofilms, which are considered a major obstacle to the ability of antibiotics to destroy bacteria [55].

Analysis of the vB_ZEFP phage genome identified a CHAP domain lysin (CDS 17) and a peptidoglycan recognition hydrolase (CDS 19) that mediate host cell lysis. Further study of the activities of these proteins could facilitate the use of these enzymes to treat bacterial infections in the future, and particularly as lysins may be more stable than phages. The activity of the vB_ZEFP phage were further tested in root canal infections using ex vivo two chamber bacterial leakage model of human teeth. Results of turbidity measurement revealed a greater ability of vB_ZEFP phage to reduce bacterial leakage from the root apex compared to other treatments. The enumeration of bacterial counts after 72 h confirmed the reduction in viable bacteria for the phage treated groups compared to conventional hypochlorite treatment. Moreover, vB_ZEFP phage proved to be effective when used in combination with hypochlorite allowing for the use of dual therapies. Our results suggest that this phage has potential for phage applications and efficacy in the prevention of infection after root canal treatments.

## Figures and Tables

**Table 1:** Antibiotic sensitivity of *E. faecalis* root canal isolates.

Antibiotics	β-Lactams		Aminoglycosides		Quinolones		Tetracycline		Macrolides	Streptogramins	Glycopeptide	Oxalidinone	Macrobid	MAR Index
	PEN G	AMP	GEN	STR	CIP	LVX	TET	TGC	ERY	Q-D	VAN	LZD	NIT	
EF.1	S	S	S	S	S	S	S	S	S	S	S	S	S	0
EF.2	S	S	R	R	R	R	R	S	R	R	S	S	S	0.54
EF.3	S	S	S	S	S	S	S	S	S	S	S	S	S	0
EF.4	R	R	R	R	R	R	R	S	R	R	S	S	R	0.77
EF.5	R	R	S	S	R	R	S	S	S	S	S	S	S	0.28
EF.6	S	S	R	R	R	R	R	S	S	R	S	S	S	0.42
EF.7	S	S	S	S	S	S	S	S	S	S	S	S	S	0
EF.8	R	R	S	S	R	R	R	S	R	R	S	S	S	0.5
EF.9	R	S	R	R	S	S	S	S	R	S	S	S	S	0.28
EF.10	S	S	S	R	R	R	S	S	R	R	S	S	S	0.35
EF.11	R	S	S	S	S	S	R	S	R	S	S	S	S	0.21
EF12	R	S	S	R	R	R	S	S	S	S	S	S	R	0.35
EF.13	s	S	R	S	R	R	S	S	S	R	S	S	S	0.28
EF.14	S	S	R	S	S	S	R	S	R	R	S	S	R	0.35
EF.15	S	S	S	S	S	S	S	S	S	S	S	S	S	0
EF.16	R	R	R	R	S	S	S	S	R	S	S	S	S	0.35
EF.17	R	S	R	S	R	S	S	S	S	S	S	S	S	0.21
EF.18	S	S	R	R	S	S	R	S	R	R	S	S	S	0.35
EF.19	R	R	S	S	R	S	S	S	S	S	S	S	S	0.28
EF.20	R	R	S	S	R	R	R	S	S	S	S	S	R	0.42
Total R %	10 (50%)	6 (30%)	9 (45%)	8 (40%)	11 (55%)	9 (45%)	8 (40%)	0	9 (45%)	8 (40%)	0	0	4 (20%)	

Abbreviations are as follows: (R): Resistant (S): sensitive, MAR: Multi- drug resistant. PEN G: Benzylpenicillin G, AMP: Ampicillin, GEN: Gentamycin High Level (Synergy), STR: Streptomycin High Level (Synergy), CIP: Ciprofloxacin, LVX: Levofloxacin, TET: Tetracyclin, TGC: Tigecyclin, ERY: Erythromycin, Q-D: Quinopristin⁄Dalfopristin, VAN: Vancomycin, LZD: Linezolid NIT: Nitrofurantoin. Therapeutic Interpretation Guideline:CLSI M100-S25 (2015).

**Table 2:** Phage vB_ZEFP ORFs with putative protein encoding functions.

CDS	ORF Location (nt)	Putative Function
1	172–342	hypothetical protein
2	344–712	hypothetical protein
3	786–1136	single-stranded DNA-binding protein
4	1204–1383	hypothetical protein
5	1380–1544	hypothetical protein
6	1557–1721	hypothetical protein
7	1734–1886	hypothetical protein
8	1870–2292	hypothetical protein
9	2285–3526	terminase large subunit
10	3539–5887	DNA polymerase
11	5947–6117	hypothetical protein
12	6117–6323	hypothetical protein
13	6314–6487	hypothetical protein
14	6488–6727	transcriptional regulator
15	6724–7221	HNH homing endonuclease
16	7221–7406	putative transmembrane protein
17	7406–8824	CHAP domain-containing lysin
18	8943–9389	DNA binding protein
19	9406–10182c	peptidoglycan recognition and hydrolase
20	10183–10575c	hypothetical protein
21	10575–12329c	tail fiber protein
22	12331–13119c	distal tail protein
23	13131–14696c	phosphodiesterase
24	14710–15246c	hypothetical protein
25	15296–16342c	phage capsid and scaffold
26	16358–17575c	phage capsid and scaffold
27	17577–17747c	capsid related protein
28	17842–18177c	hypothetical protein

Nucleotide locations with suffix c indicate the reading frame is located on the complementary strand.

## Data Availability

The authors declare that all data supporting the findings of this study are available within the article and its supplementary information files. The DNA sequence of bacteriophage vB_ZEFP is available in the public database GenBank under the accession number MT747434.

## Supplementary Materials

The following are available online at https://www.mdpi.com/2076-2607/9/3/517/s1, Table S1: PCR primers and products for the detection of *E. faecalis* virulence genes; Table S2: Host range of *E. faecalis* bacteriophages. Figure S1 Gel electrophoresis of PCR amplification products of *E. faecalis* virulence genes.
