# Supplementary material for: Bacteriophages to Control Multi-Drug Resistant Enterococcus faecalis Infection of Dental Root Canals
Source: Microorganisms. 2021 Mar 3;9(3):517. doi: 10.3390/microorganisms9030517 (PMC7998577; doi:10.3390/microorganisms9030517)
Supplement: Supplementary file 1 [file microorganisms-09-00517-s001.pdf]

Supplementary Table S1. PCR primers and products for the detection of *E. faecalis* virulence genes.

Agent	Virulence factor	Oligonucleotide primers sequences (5'-3')	Product size (bp)	Reference
Enterococcus faecalis	*esp*	AGATTTCATCTTTGATTCTTGG	510 bp	**Vankerckhoven *et al*., 2004**
		AATTGATTCTTTAGCATCTGG		
	gelE	TATGACAATGCTTTTTGGGAT	213 bp	
		AGATGCACCCGAAATAATATA		
	asa1	GCACGCTATTACGAACTATGA	375 bp	
		TAAGAAAGAACATCACCACGA		
	cylA	ACTCGGGGATTGATAGGC	688 bp	
		GCTGCTAAAGCTGCGCTT		
	ace	GGAATGACCGAGAACGATGGC	616 bp	**Creti *et al*., 2004**
		GCTTGATGTTGGCCTGCTTCCG		
	EF3314	AGAGGGACGATCAGATGAAAAA	566 bp	
		ATTCCAATTGACGATTCACTTC		

Supplementary Table S2. Host range of *E. faecalis* bacteriophages

Bacterial sp.	Isolates sources	ϕZEF1	ϕZEF2
E. faecalis1	Isolated by this study	+	+
E. faecalis2	Isolated by this study	+	+
E .faecalis3	Isolated by this study	-	-
E. faecalis4	Isolated by this study	+	+
E .faecalis5	Isolated by this study	+	+
E .faecalis6	Isolated by this study	-	-
E. faecalis7	Isolated by this study	+	+
E. faecalis8	Isolated by this study	+	+
E. faecalis9	Isolated by this study	+	+
E. faecalis10	Isolated by this study	+	+
E. faecalis11	Isolated by this study	-	-
E. faecalis12	Isolated by this study	+	+
E. faecalis13	Isolated by this study	+	+
E. faecium	Animal Health Research Institute, Dokki, Egypt.	-	-
S. mutans	Cairo MIRCEN reference (Gene bank accession number : Z95910)	-	-
E. gallinarum	Animal Health Research Institute, Dokki, Egypt.	-	-
S. aureus	Faculty of Science, Zagazig University, Egypt (Gene Bank accession number: KR270348).	-	-
E. coli	Department of Zoonoses, Faculty of Veterinary Medicine, Zagazig University.	-	-
P. aeruginosa	Faculty of Science, Zagazig University, Egypt (Gene Bank under accession number: LC514698).	-	-

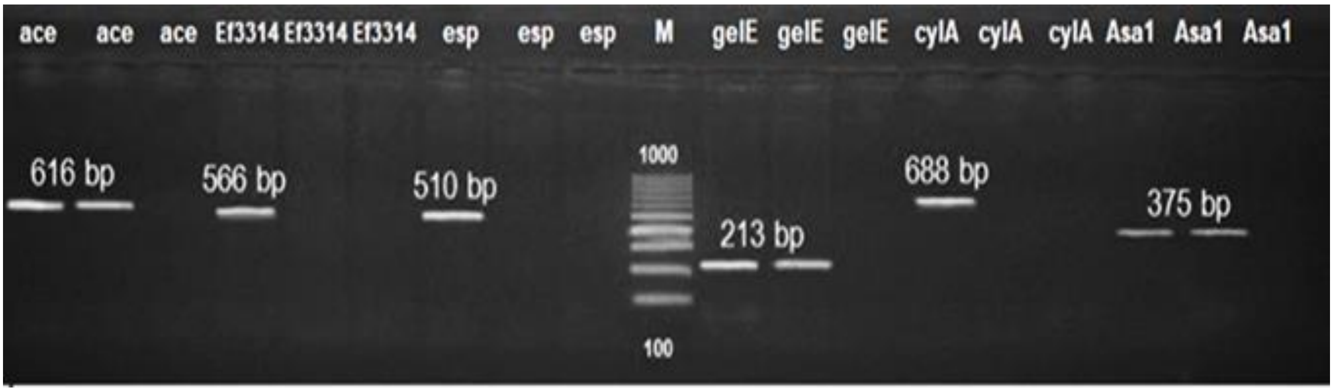

Supplementary Figure S1. Gel electrophoresis of PCR products of virulence genes amplified from *Enterococcus* isolate No. 4. PCR amplification of ace, EF3314, esp, gelE, cylA and asa1 genes genes at 616, 566, 510, 213, 688 and 375 bp, respectively. Presented from left to right: ace gene (lane 1, positive control, lane 2, positive isolate, lane 3, negative control); EF3314 gene (lane 4, positive control, lane 5, negative isolate, lane 6, negative control), esp gene (lane 7, positive control, lane 8 negative isolate, lane 9, negative control); lane 10, (M) 100 bp DNA Ladder; gelE gene (lane 11, positive control, lane 12, positive isolate, lane 13, negative control); cylA gene (Lane 14, positive control, lane 15, negative isolate, lane 16, negative control); asa1 gene (lane 17, positive control, lane 18, positive isolate, lane 19, negative control).
